# Genetic and environmental regulation of arbuscular mycorrhizal responsiveness in *petunia*: Implications for breeding and trait selection

**DOI:** 10.1111/plb.70185

**Published:** 2026-01-28

**Authors:** J. Brandes, R. Halitschke, K. Fischer, I. T. Baldwin, P. Franken

**Affiliations:** ^1^ Erfurt Research Centre for Horticultural Crops (FGK), Erfurt University of Applied Sciences Erfurt Germany; ^2^ Institute of Microbiology, Friedrich Schiller University Jena Germany; ^3^ Department of Molecular Ecology Max Planck Institute for Chemical Ecology Jena Germany; ^4^ Leibniz Institute of Vegetable and Ornamental Crops Großbeeren Germany

**Keywords:** arbuscular mycorrhizal fungi, blumenol, GxE Interaction, light intensity, mycorrhizal responsiveness, *Petunia axillaris*, *Petunia exserta*, *Petunia hybrida*, *Petunia inflata*, phosphate, photoperiod, *Rhizoglomus irregulare*

## Abstract

- Arbuscular mycorrhizal (AM) fungi enhance plant nutrition and stress tolerance, yet their agricultural use remains limited because symbiotic outcomes are unpredictable. Mycorrhizal responsiveness (AM‐responsiveness)—the host's growth response to AMF inoculation—offers a potential breeding target.
- We investigated variation in AM‐responsiveness among *Petunia hybrida*, *P. axillaris*, *P. exserta* and *P. inflata*, and explored its genetic and environmental determinants. Plants were inoculated with *Rhizoglomus irregulare* and analysed for biomass, AMF colonization, phosphate uptake, phosphate transporter expression and accumulation of the foliar biomarker 11‐carboxyblumenol C‐glucoside.
- Species differed strongly in colonization intensity, biomass and biomarker accumulation. Based on contrasting AM‐responses between *P. axillaris* and *P. exserta*, a recombinant inbred line (RIL) population derived from these parents was used to assess AM‐responsiveness as a quantitative trait under variable environmental conditions. The RILs showed transgressive segregation for biomass responses, confirming a heritable component, while strong genotype × environment (G × E) interactions demonstrated environmental dependency.
- These results highlight AM‐responsiveness as a genetic trait suitable for breeding but emphasize the need to account for environmental variation. Foliar blumenols proved effective non‐destructive indicators of colonization, supporting their potential in high‐throughput screening for mycorrhizal traits.

## Graphical abstract

Arbuscular mycorrhiza responsiveness varies strongly among *Petunia* species and RILs, representing a genetically determined trait with breeding potential that is nonetheless strongly modulated by environmental conditions.

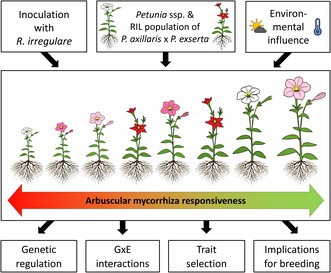

## INTRODUCTION

The arbuscular mycorrhizal (AM) symbiosis is an integral part of most terrestrial ecosystems, providing essential ecosystem services that support plant nutrition and plant health (Gianinazzi *et al*. 2010; Burow *et al*. 2021). Despite decades of research and its recognized agronomic potential (Bitterlich *et al*. 2020), adoption in agriculture and horticulture remains limited because outcomes are unpredictable and range from negative to strongly positive effects on plant growth (Stahlhut *et al*. 2023). Beyond growth, AMF can alter metabolite profiles relevant to taste and human health, affecting product quality (Schroeder *et al*. 2012; Pasković *et al*. 2021). Where a given plant–fungus interaction falls on the mutualism–parasitism continuum (Johnson *et al*. 1997) depends on fungal identity, environment and especially host genotype (Kandhasamy *et al*. 2020; de Souza Campos *et al*. 2021). These factors motivate treating mycorrhizal responsiveness (AM‐responsiveness)—the host's growth response to AMF inoculation—as a quantitative, genetically determined trait and potential breeding target, ideally supported by molecular markers linked to genes or quantitative trait loci (QTL).

There is already a large body of work on the molecular basis of the AM symbiosis. Basically, two approaches have been pursued. On the one hand, genes that play a role in the early phase of the symbiosis were identified by studying mutants that no longer form mycorrhiza (Gutjahr & Parniske 2013). On the other hand, transcriptome analyses were used to identify differentially expressed genes (Vangelisti *et al*. 2018), whose function was subsequently characterized by targeted interventions in their expression (e.g. Krajinski *et al*. 2014). These were mostly genes that play a role in the later phase of the symbiosis and are often involved in nutrient exchange between the symbiotic partners. Silencing these genes can lead to changes in both AMF root colonization and AM‐responsiveness (Bitterlich *et al*. 2014). A third approach via QTL mapping for AM‐responsiveness has only rarely been followed. QTL have been found among recombinant inbred lines (RILs) in maize (Kaeppler *et al*. 2000) and in crosses between two *Allium* species (Galván *et al*. 2011). In the family of Solanaceae, eight QTL were detected in a RIL population derived from wild species *Solanum pimpinellifolium*, but this study only investigated fungal colonization (Plouznikoff *et al*. 2019). In addition, genome‐wide association studies (GWAS) mapped regions in the genome of wheat (Lehnert *et al*. 2017) and durum wheat (de Vita *et al*. 2018) involved in positive responses to AMF inoculation. However, follow‐up studies to identify the corresponding genes or to develop molecular markers for breeding based on these QTL have not been published. Importantly, none of these studies evaluated the environmental impact on AM‐responsiveness, especially in relation to light intensity and photoperiod. Although Ramírez‐Flores *et al*. (2020) and Li *et al*. (2023) found QTL for AM‐responsiveness in maize linked to yield and nutritional host plant status, both trials were conducted during a single season and therefore did not account for environmental variation. Lehnert *et al*. (2017) conducted a two‐year greenhouse trial to perform GWAS in wheat but kept light conditions nearly constant, minimizing photoperiodic differences. Galván *et al*. (2011) performed a two‐year greenhouse trial and analysed QTL for each year individually, revealing only partial overlap between years.

Several studies have focused on the dependency of AMF colonization and AM‐responsiveness on environmental factors such as light intensity and photoperiod. Reduced light intensity negatively impacts both AMF colonization and plant responsiveness. In tomato, stomatal conductance and net photosynthesis decreased under low light, reducing or negating AMF benefits (Bitterlich *et al*. 2019). In lettuce, AMF colonization under low light was markedly reduced, along with a decrease in vesicle formation (Pozo de la Hoz *et al*. 2021). Light quality, especially the ratio of red to far‐red light (R:FR), affects root colonization and influences growth and defence (Saha *et al*. 2022). Janos (2007) demonstrated that short photoperiods and reduced light intensities significantly decrease root colonization, thereby diminishing the beneficial impacts of mycorrhiza. These results underscore the importance of AM‐responsiveness as a breeding target but also illustrate the difficulties caused by environmental influences, in particular the light dependence of AMF and thus the differences in AM‐responsiveness among plants (Konvalinková & Jansa 2016; Lutz *et al*. 2023).

Blumenols, a class of C_13_ apocarotenoids, play a prominent role in AMF colonization of host roots. These metabolites accumulate specifically in root cortical cells harbouring arbuscules and are translocated to shoots, allowing quantification of colonization intensity (Wang *et al*. 2018). Because foliar blumenol quantification is non‐destructive and scalable, it has great potential for breeding or screening genotypes with improved AMF associations (Wang *et al*. 2018; Fiorilli *et al*. 2019). In *Nicotiana attenuata*, foliar blumenol concentrations correlate positively with lipid transfer to AMF (You *et al*. 2023). In rice, Servanté *et al*. (2024) demonstrated a strict correlation between leaf blumenol C‐glucoside levels and root colonization, confirming that blumenol accumulation is tightly linked to arbuscule formation. However, high blumenol levels do not always predict proportionally high AM‐responsiveness, since the growth benefit depends on environmental factors such as nutrient status or plant–AMF compatibility (Kaur *et al*. 2022).

*Petunia*, an annual bedding crop with a high diversity in colour and morphology, is not only of great economic importance for ornamental production but also a valuable model plant (Gerats & Vandenbussche 2005). *Petunia* has been used to investigate mycorrhizal symbiosis since the first studies were published (Reddy *et al*. 2007). It offers a versatile genetic toolbox (Druege & Franken 2019), including a wide variety of genotypes from wild species to commercial cultivars. *Petunia hybrida* cv. ‘Mitchell’ (W115) has been widely used for mycorrhizal research, for example on phosphate repression of mycorrhization (Breuillin *et al*. 2010) and mycorrhiza‐induced resistance (Hayek *et al*. 2014). The *dTPH1* transposon system in line W138 (Vandenbussche *et al*. 2008) allows forward and reverse genetic approaches to study genes involved in mycorrhiza establishment and function (Wegmüller *et al*. 2008; Rich *et al*. 2015). Modern cultivars and lines originate from crosses between wild species (Strazzer *et al*. 2023). Several recombinant inbred line (RIL) populations based on crosses between *P. axillaris*, *P. exserta* and *P. inflata* (syn. *P. integrifolia* subsp. *inflata*; Govaerts *et al*. 2021) have been established. Guo *et al*. (2017) characterized two RIL populations using genotyping‐by‐sequencing, detecting QTL related to crop timing and quality traits (Cao *et al*. 2018, 2019). Additionally, a *P. axillaris × P. exserta* population revealed QTL for flowering and performance traits under different environments (Chen & Warner 2022).

The aim of this study was to determine whether *Petunia* can be used to identify genetic factors of AM‐responsiveness despite environmental influences. This could lead to the discovery of markers associated with relevant QTLs or genomic regions useful for breeding. In addition, we examined how environmental conditions—especially light intensity and photoperiod—affect AM‐responsiveness. The AM‐responsiveness of three wild *Petunia* species (*P. axillaris*, *P. exserta*, *P. inflata*), together with *P. hybrida* cv. ‘Mitchell’ and the *dTPH1* insertion line W138, was tested with respect to root colonization, growth, phosphate uptake, phosphate transporter expression and blumenol accumulation. Based on these results, the RIL population derived from *P. axillaris × P. exserta* was chosen for a second trial to assess the quantitative nature of AM‐dependent growth response. In a third trial, this population was cultivated under three environmental conditions differing in light intensity and photoperiod to evaluate the environmental dependency of AM‐responsiveness.

## MATERIALS AND METHODS

### Plant cultivation and application of *Rhizoglomus irregulare* inoculum

In the first trial, seeds of four *Petunia* species (*P. hybrida* cv. ‘Mitchell’, *P. axillaris*, *P. exserta*, and *P. inflata*), as well as the high‐copy number *dTPH1* transposon insertion line W138 were surface‐sterilized and germinated in Petri dishes filled with half‐strength Murashige & Skoog medium supplemented with vitamins (Duchefa Biochemie B.V., Haarlem, The Netherlands) for 14 days under a 16/8‐h photoperiod at temperatures of 24°C (day) and 22°C (night). To acclimate the seedlings to greenhouse conditions, they were transferred to multitrays with nutrient‐poor soil and grown under hoods for 2 weeks. The plantlets were then planted in 1000 cm^3^ pots filled with a mixture of sand and vermiculite (1:1) and inoculated with either *R. irregulare* QS 81 inoculum (INOQ GmbH, Soltau, Germany) or a mock inoculum containing only the carrier material (Hayek *et al*. 2012). To confirm that the observed growth effect of the plants was attributable to *R. irregulare* and not to other microorganisms present in the inoculum, 100 mL of inoculum per pot was filtered through a 40 μm sieve with 100 mL of water. Thereafter, the filtrate was used to water the control plants, as the filtrate was free of *R. irregulare* but still contained other microorganisms, allowing for the attribution of changes in plant growth exclusively to *R. irregulare*. The experiment was conducted for 5 weeks in a greenhouse chamber under natural light conditions from May to June (Table 1). The experimental design was completely randomized including five replicates per species and mycorrhizal treatment resulting in 50 plants in total. The plants were fertilized with a modified Hoagland solution with 10% of the optimal phosphate amount (Hayek *et al*. 2012) and irrigated daily as needed.

**Table 1:** Environmental conditions of all trials.

environmental conditions	trial 1	trial 2	trial 3		
			experiment 1	experiment 2	experiment 3
average temperature	23.9°C	22.2°C	22.6°C	23.3°C	18.8°C
average relative humidity	43.6% rH	63% rH	51.4% rH	49.5% rH	50.6% rH
average photoperiod	16 h	13 h	16 h	14 h	11 h
average light intensity	104.8 μmol m^−2^ s^−1^	215.6 μmol m^−2^ s^−1^	391.7 μmol m^−2^ s^−1^	419.9 μmol m^−2^ s^−1^	260 μmol m^−2^ s^−1^

In the second trial, a subset consisting of 19 randomly selected recombinant inbred lines (RILs) from a RIL population derived by *P. axillaris* × *P. exserta* (Warner & Walworth 2010) including their parents was grown for 9 weeks in five replicates per genotype and treatment resulting in 210 plants in total. The plants grew for 9 weeks and were treated identically to those in the first trial except for the environmental conditions, as the plants were cultivated in a greenhouse from March to May (Table 1).

In the third trial, the same 19 RILs including *P. axillaris* and *P. exserta* were cultivated in five replicates per genotype and AMF treatment three times within 1 year. In each of the three experiments, the plants grew for 6 weeks and were treated equally to the first trial except for the environmental conditions (Table 1).

### Harvest

In the first trial, the plants in their generative phase were harvested 5 weeks after inoculation. During the harvest, plant height was measured, the numbers of generative organs (total number of buds, flowers and seed capsules) were counted, and the plants were cut at the stem base. Fresh weight of the shoot was measured, and the leaves were picked, counted, and measured separately. Then, the stem weight was recorded. To analyse blumenol derivatives, two samples of 200 mg of fresh leaf tissue per plant were taken. The root system was carefully removed from the substrate by shaking and washing in tap water; afterwards, root fresh weight and root length of the whole root apparatus were measured. Samples for RNA extraction were collected, immediately frozen in liquid nitrogen, and samples for microscopic analysis were stored in 10% ethanol. After drying the tissues for 24 h at 80°C, the stem, leaf and root dry weight were determined.

In the second trial, the plants which also were in their generative phase were harvested 9 weeks after inoculation. Due to the high number of plants, fewer parameters were evaluated. As stem and leaves were not separated during harvest, only two parameters for above ground biomass (shoot fresh weight, shoot dry weight) were measured. Root fresh and dry weight were evaluated, but root length was not measured.

In the three experiments of the third trial, the plants were measured and harvested the same way as in the first trial. After measuring shoot length and complete plant fresh weight, the leaves were separated from the stem and each weight was measured individually. After washing of the root system, samples for microscopic analysis were taken and root fresh weight was measured. The leaf, stem, and root samples were dried for 24 h and measured afterwards.

### Microscopic analysis and phosphorus measurement

According to Vierheilig *et al*. (1998), root samples were treated with a 10% KOH solution for 60 min at 80°C, then washed three times with tap water and incubated in 0.1 M HCl for 5 min. The roots were then incubated overnight in a vinegar‐ink solution. After staining, the roots were washed three times with tap water and stored in tap water. For the first and the third trial, the stained roots were cut into 1 cm pieces, and 30 root pieces per replicate were analysed using the method described by Trouvelot *et al*. (1986). The mycorrhizal colonization rate including the abundance of arbuscules and vesicles was assessed using the INOQ Calculator Advanced (Mercy 2017). For the second trial, only the percentage of fungal root colonization was assessed by the grid‐line intersection method (McGonigle *et al*. 1990). In addition, vesicle abundance was evaluated using a scale reaching from 0 (no vesicles) to 4 (very high number of vesicles). For phosphorus analysis, dried leaf samples were ground, and the phosphorus content was measured using ICP‐OES according to the DIN EN 15621:2017–10 norm by the Thüringisches Landesamt für Landwirtschaft and Ländlichen Raum (Jena, Germany).

### RNA extraction and quantitative RT‐PCR

Frozen root samples from the first experiment were ground at −10°C using a homogenizer (Precellys), and RNA was extracted using the RNeasy Plant Mini Kit (Qiagen, Hilden, Germany). The cDNA was synthesized using the QuantiTect Reverse Transcription Kit (Qiagen) and was used to assess the gene activity of the mycorrhiza‐specific phosphate transporter gene *PhPT4* and the mycorrhiza‐enhanced phosphate transporter gene *PhPT5* by qRT‐PCR according to Wegmüller *et al*. (2008) and Tan *et al*. (2012). The Δ_Ct_ method, using *PhGapDH* as a housekeeping gene, was applied to obtain relative expression values (Pfaffl 2001).

### Targeted analysis of blumenol derivatives

Frozen leaf tissue from the first experiment was weighted, and the blumenol derivatives 11‐carboxyblumenol C‐Glc, 11‐hydroxyblumenol C‐Glc and blumenol A‐Glc were extracted using the method described by Mindt *et al*. (2019). Extracts were analysed by UHPLC‐triple quadrupole MS/MS with multiple‐reaction‐monitoring (MRM) settings optimized for each compound. The tissue concentration of the blumenol derivatives was quantified based on the deuterated abscisic acid (D_6_‐ABA) internal standard, which was added during the extraction process.

### Statistical analysis

Relative trait responses were calculated for each genotype based on the mean values of AMF‐inoculated (Myc) and uninoculated control (K) plants. For each trait and experimental replicate, the relative mycorrhizal response was expressed as a percentage change relative to the control treatment: Relative response (%) = [(Myc − K)/K] × 100 resulting in 25 relative datapoints based on means of five biological replicates per genotype. Data from qPCR, phosphorus analysis, blumenol measurements and absolute and relative harvest traits from Trials 1 and 2 were first tested for equality of variances using Levene's test (Levene 1961; Field *et al*. 2012). Depending on the result of this test, analysis of variances (ANOVA) assuming equal or unequal variances was conducted. Post hoc analysis of data with equal variances was performed using Tukey's Test at *p* ≤ 0.05, and data with unequal variances was analysed using Dunnett's test at *p* ≤ 0.05 or for pairwise comparisons using Mann–Whitney *U*‐test at *p* ≤ 0.05 (Mann & Whitney 1947). Correlations were calculated using Pearson correlation. The fungal colonization rates were calculated using the RAMF package (Chiapello *et al*. 2019). To evaluate the effects of genotype (*G*), environment (*E*) and their interaction (*G × E*) on AMF colonization and relative harvest traits of the third trial, a linear modelling approach was applied for each colonization and relative harvest trait. The statistical model used was: Trait ∼ Genotype + Experiment + Genotype × Experiment. *Genotype* refers to the specific RIL or parental accession, and *Experiment* represents the environmental treatment corresponding to the three independent experimental runs. Prior to analysis, data were tested for normality and homoscedasticity. In cases of missing values, group means (within each *Genotype × Experiment* combination) were used for imputation. Type‐III analysis of variance (ANOVA) was conducted using the Anova() function from the car package in ‘R’.

Genetic similarity between each recombinant inbred line (RIL) and *Petunia axillaris* was calculated based on a bi‐allelic marker dataset comprising 368 bin markers (Guo *et al*. 2017). Each marker position was scored as carrying either the *P. axillaris* allele (‘a’) or the *P. exserta* allele (‘b’). For each RIL, the proportion of loci carrying the *P. axillaris* allele among all informative loci (i.e. those with allele calls ‘a’ or ‘b’) was determined using the following formula: % similarity = (number of ‘a’ alleles)/(number of ‘a’ + ‘b’ alleles) × 100. Markers with missing or ambiguous calls were excluded. The genotype matrix was transposed such that each RIL corresponded to a row, and similarity values were computed using ‘R’. The resulting percentage of genetic similarity to *P. axillaris* was used as a continuous explanatory variable in correlation analyses with relative harvest data derived from the three independent experiments of the third trial.

Principal component analyses (PCA) were performed in ‘R’. Graphics were edited using ‘Excel’ and ‘Inkscape’.

## RESULTS

### AMF‐dependent growth responses of different *petunia* genotypes and a recombinant inbred line population

**Fig. 1:**
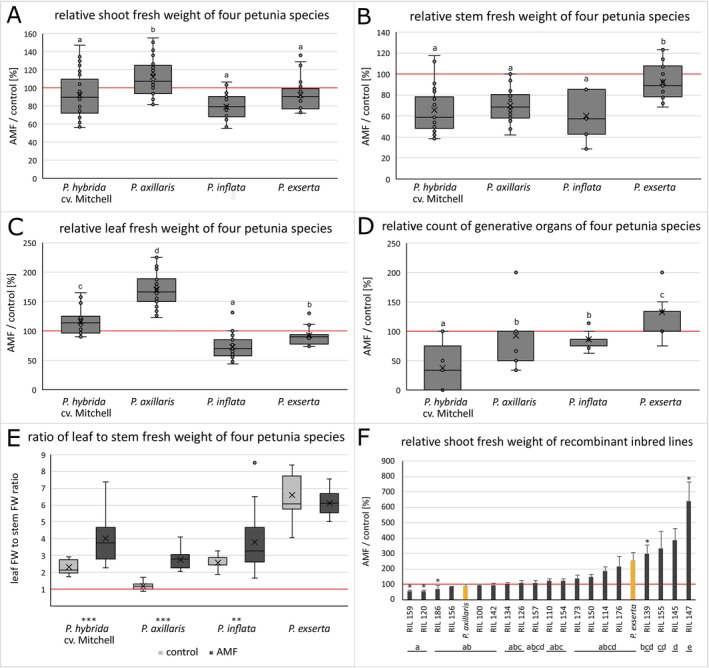
Relative shoot harvest data (ratios of AMF‐inoculated to control plants) including shoot fresh weight (A), stem fresh weight (B), leaf fresh weight (C), the number of generative organs (D) and the ratio of leaf to stem fresh weight (E) of four *Petunia* species 5 weeks after inoculation with *R. irregulare* (Trial 1) and the shoot fresh weight (F) of 19 recombinant inbred lines 9 weeks after inoculation with *R. irregulare* (Trial 2). The red line indicates no differences between mycorrhizal and control plants (100%). Different letters indicate significant differences (*p* ≤ 0.05) between the genotypes using Dunnett's test (A, F) or Tukey's test (B, C, D). Asterisks indicate significant differences (* ≤ 0.05, ** ≤ 0.01, *** ≤ 0.001) between control and AMF‐inoculated plants using Mann–Whitney‐*U*‐test (E, F). The number of biological replicates was n = 5 for all panels (A–F). Error bars in panel F represent the standard error of the mean (SEM).

To evaluate the impact of *Rhizoglomus irregulare* inoculation, growth parameters were assessed 5 weeks after inoculation in Trial 1 and 9 weeks after inoculation in Trial 2. Since the transposon insertion line W138 showed no significant differences between control and AMF‐inoculated plants (Table S1), it was excluded from Fig. 1 and Figs. S1 and S3.

Across the four species, relative shoot fresh weight was only marginally influenced by AMF inoculation, with *P. axillaris* differing significantly from *P. hybrida*, *P. exserta* and *P. inflata* (Fig. 1A). In Trial 2, 19 RILs from *P. axillaris × P. exserta* and their parents showed a gradient in relative shoot fresh weight (Fig. 1F). Five RILs responded significantly to AMF, whereas neither parent differed between control and AMF treatment. ANOVA revealed significant genotypic differences (Fig. 1F, letters). The three RILs with the largest positive AMF effects (RIL 155, 145, 147) differed significantly from *P. axillaris*; only RIL 147 also differed from *P. exserta*.

Relative stem fresh weight decreased across all species, least in *P. Exserta* (Fig. 1B). Relative leaf fresh weight varied strongly (Fig. 1C): *P. axillaris* showed a pronounced increase, *P. hybrida* a slight increase, while *P. exserta* and *P. inflata* produced less leaf biomass when inoculated with AMF. The number of generative organs (buds + flowers + capsules) also varied (Fig. 1D). Mycorrhization delayed flowering in *P. hybrida* but accelerated it in *P. exserta*.

AMF altered biomass partitioning (Fig. 1E). *P. hybrida*, *P. axillaris* and *P. inflata* developed higher leaf:stem ratios after inoculation, while *P. exserta* remained unchanged. In case of *P. hybrida*, this increase was caused by a decrease in relative stem fresh weight (Fig. 1B) combined with no changes in relative leaf fresh weight (Fig. 1C). For *P. axillaris*, the large increase in leaf:stem ratio was due to a decrease in stem fresh weight (Fig. 1B) in combination with a large increase in leaf fresh weight (Fig. 1C). *P. inflata* also showed an increase in leaf:stem ratios, mainly due to a greater decrease in stem fresh weight than in leaf fresh weight.

Root fresh weight decreased by 40%–60% in all four species, but root length was unaffected (Fig. S1A,B). *P. axillaris* differed significantly from the other wild species *P. inflata* and *P. exserta* (Fig. S1A). In Trial 2, no significant difference in root fresh weight occurred between parental species; three RILs (145, 176, 147) differed significantly from *P. axillaris*, and only RIL 147 differed from *P. exserta* (Fig. S1C). Neither *P. axillaris* nor *P. exserta* developed significantly different root fresh weight after AMF treatment compared to control plants (Fig. S1C, asterisks).

### AMF colonization dynamics across different genotypes and experiments

**Fig. 2:**
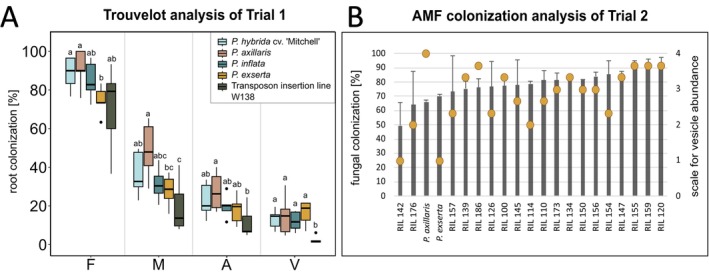
Root colonization with *R. irregulare* of five *Petunia* genotypes of Trial 1 (A) and a RIL population of Trial 2 (B). A: Root colonization analysis performed using Trouvelot analysis. F = frequency of AMF in the root system, M = intensity of AMF in the root system, A = arbuscule abundance in the root system, V = vesicle abundance in the root system. Different letters indicate significant differences between samples (*p* ≤ 0.05) according to Kruskal–Wallis test without correction method. B: Root colonization of 19 recombinant inbred lines, *P. axillaris* and *P. exserta* performed using a simplified scoring method. Vesicle abundance is displayed as orange dots ranging from very low abundance (1) to very high abundance (4), n = 3, error bars in panel B represent the standard deviation (SD).

Colonization rates and patterns of *R. irregulare* were assessed using the Trouvelot method for Trial 1 and Trial 3 (Fig. 2A; Fig. S2) and a simplified scoring method in Trial 2 (Fig. 2B). In Trial 1, colonization frequency (F) exceeded 70 % in all species, reaching nearly 100 % in *P. axillaris* but only moderate levels in *P. exserta* (Fig. 2A). Colonization intensity (M) followed the same pattern. Arbuscule abundance (A) differed significantly between *P. axillaris* and W138, which showed almost no vesicles (V). Control roots were AMF‐free, confirming no cross‐contamination.

In Trial 2, mean AMF colonization ranged from 50% to 100%, averaging about 80% among RILs (Fig. 2B). Most RILs were more colonized than their parents. Vesicle abundance was lowest in RIL 142 and *P. exserta* (score 1) and highest in *P. axillaris* (score 4).

Trial 3, performed under three environmental regimes, revealed substantial genotype × environment variation (Fig. S2). Some genotypes exhibited consistent colonization patterns, whereas others showed pronounced environmental sensitivity. Mean frequency of colonization of all RILs was highest in Experiment 2, followed by Experiment 1, while mean F% of Experiment 3 was considerably lower. Colonization frequency, intensity and arbuscule and vesicle abundances varied significantly across experiments, being consistently lowest in Experiment 3 with shorter photoperiod and reduced light.

### Genetic and environmental determinants of AM‐responsiveness

**Fig. 3:**
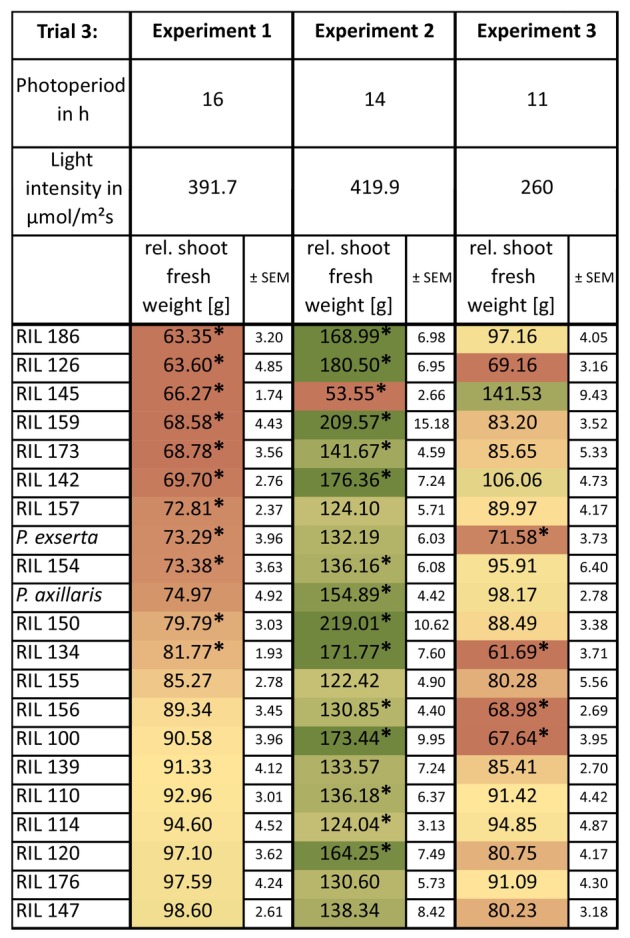
Relative shoot fresh weight (ratios of AMF‐inoculated to control plants) of Trial 3, consisting of Experiment 1, 2, and 3. Results were sorted according to the AM‐responsiveness of Experiment 1. Significant differences between mock and AM‐inoculated plants were calculated by a permutation test and are indicated by asterisks (*p* ≤ 0.05); the number of biological replicates was n = 5 for all three experiments.

Environmental effects on AM‐responsiveness were analysed in the *P. axillaris × P. exserta* RIL subset across three contrasting light regimes (Table 1). Relative shoot fresh weight served as an indicator of AM‐responsiveness (Fig. 3). The quantitative character of this trait is visible as a gradient ranging from a negative to a positive relative shoot weight. In Experiments 1 and 3, relative values were below 100%, indicating negative responsiveness, whereas Experiment 2 showed positive values (>100%) for nearly all RILs, indicating an overall, genotype‐independent, positive AM‐responsiveness in a certain light regime.

**Fig. 4:**
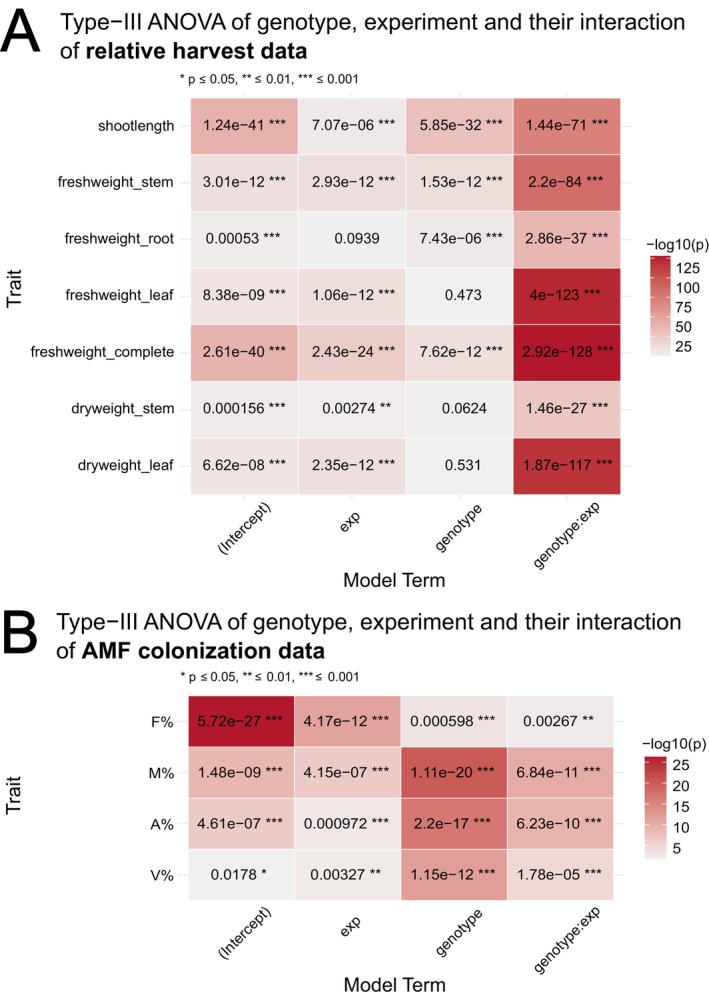
Significance matrix for the effects of genotype, environment (exp), and their interaction on relative harvest data (A) and AMF colonization traits (B) of Trial 3. Each cell shows the p‐value and significance level from a Type III ANOVA (linear model). The colour intensity represents the strength of significance as –log_10_(*p*), significance levels: *P ≤ 0.05, **P ≤ 0.01, ***P ≤ 0.001, n = 5.

Type III ANOVA confirmed highly significant effects of genotype, environment, and their interaction (G × E) on relative growth traits (Fig. 4A) and colonization traits (Fig. 4B). Relative growth parameters, including shoot length and biomass traits, were significantly influenced by genotype, environment, and their interaction, emphasizing the complexity of AM‐responsiveness. For several relative harvest traits, notably freshweight_leaf, dryweight_stem, and dryweight_leaf, the genotype main effect was not significant, while environment and especially genotype‐by‐environment interaction effects are highly significant. This pattern indicates that for these traits, phenotypic differences among genotypes are minor, and trait variation was mainly explained by environmental conditions and genotype‐specific responses to the environment. By contrast, traits such as shoot length and freshweight_complete display highly significant genotype effects in addition to strong environment and interaction effects, highlighting substantial genetic control as well as environmental responsiveness (Fig. 4A). For colonization parameters (F%, M%, A%, V%), all factors were highly significant, with most *p*‐values far below 0.001. The strength of the genotype effect differs among parameters, being strongest for M% and A%, and weakest (but still highly significant) for F%.

**Fig. 5:**
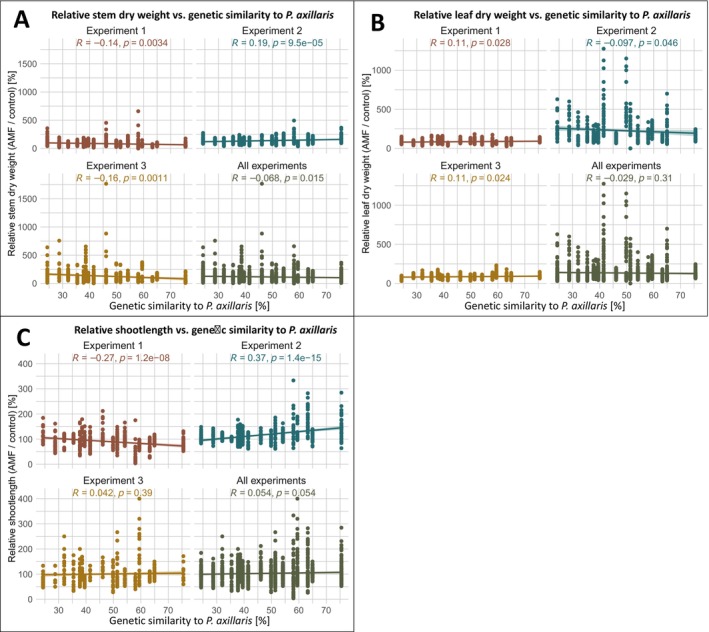
Relative harvest data in relation to genetic similarity to *Petunia axillaris*. Scatterplots showing the relationship between the relative stem dry weight (A), relative leaf dry weight (B), and relative shoot length (C) in response to arbuscular mycorrhizal fungi (AMF) and the percentage of genetic similarity to *Petunia axillaris* across recombinant inbred lines (RILs). Each panel represents one of three independent experiments or the combined dataset (‘all’) of Trial 3. Relative shoot length response was calculated as the percentage change in AMF‐inoculated plants compared to non‐inoculated controls. Genetic similarity was estimated based on the proportion of *P. axillaris* alleles across all informative marker loci. Coloured lines represent linear regressions per experiment, with corresponding Pearson correlation coefficients (r) and significance values (*p*) indicated within each panel.

Genetic similarity between each RIL and *P. axillaris* (based on 368 bin markers; Guo *et al*. 2017) was correlated with relative harvest traits (Fig. 5). Stem dry weight responses correlated weakly but significantly negatively with *P. axillaris* similarity in Experiments 1 and 3, indicating a reduction of stem dry weight after inoculation with AMF, among genotypes genetically more similar to *P. axillaris*. Although the correlation was significantly positive in Experiment 2, the overall trend within all three experiments of Trial 3 together (all) was still significantly negative, reinforcing the environment‐dependent nature of this relationship (Fig. 5A). Leaf dry weight correlations of Experiments 1 and 3 are significant, but not strongly positive, while relative dry weight of Experiment 2 was significantly negatively correlated to the similarity of *P. axillaris* suggesting a higher relative dry weight of RILs less similar to *P. axillaris* (Fig. 5B). Shoot length correlations (Fig. 5C) were significantly negative in Experiment 1, but significantly positive in Experiment 2, confirming environment‐dependent genotype performance.

**Fig. 6:**
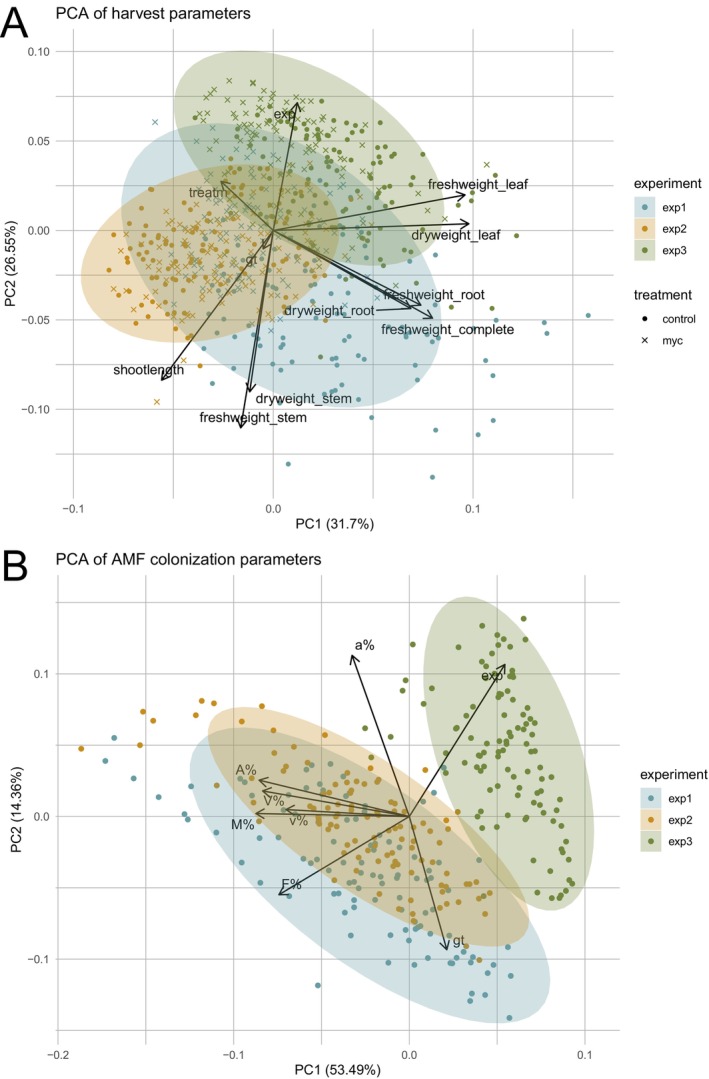
Principal component analysis (PCA) of harvest parameters under mycorrhizal and control conditions (A) and AMF colonization parameters (B) of Trial 3. A: PCA biplot based on standardized harvest traits from both mycorrhizal and non‐mycorrhizal plants. Samples are coloured by experimental replicate (exp1–exp3) and shaped by treatment (control vs. Myc). B: PCA biplot based on root colonization data from mycorrhizal plants only. Samples are coloured by experimental replicate (exp1–exp3), and ellipses represent 95% confidence intervals for each group. Loadings (arrows) indicate the contribution of individual colonization traits to the principal components, n = 5.

Principal component analysis (PCA) was performed to explore the structure of variation in both (absolute) harvest traits across three experimental replicates (Fig. 6A) and AMF colonization parameters (Fig. 6B). For harvest traits, PC1 (31.7%) reflected biomass traits; PC2 (26.6%) reflected shoot length and stem weight. Importantly, PC1 partially separated control and mycorrhizal samples, indicating a consistent treatment effect across experiments. The treatment (treatm) variable loaded negatively on PC1, opposing traits like fresh weight_leaf, suggesting that mycorrhization was generally associated with increased biomass. Ellipses revealed considerable overlap among the experimental replicates, but distinct tendencies in trait expression remained visible. Colonization PCA separated experiments clearly: PC1 (53.5%) captured overall colonization intensity, PC2 (14.4%) captured experiment‐specific variation. Notably, the variable a% (arbuscule frequency in colonized root fragments) contributed predominantly to PC2, suggesting it captures additional, experiment‐specific colonization dynamics. Interestingly, the genotype variable (gt) contributed strongly to the variance in colonization traits (Fig. 6B), but showed only marginal influence on harvest traits (Fig. 6A), indicating that colonization is more host‐controlled while biomass is more environment‐driven.

### Blumenol derivatives as shoot markers associated with AMF colonization

**Fig. 7:**
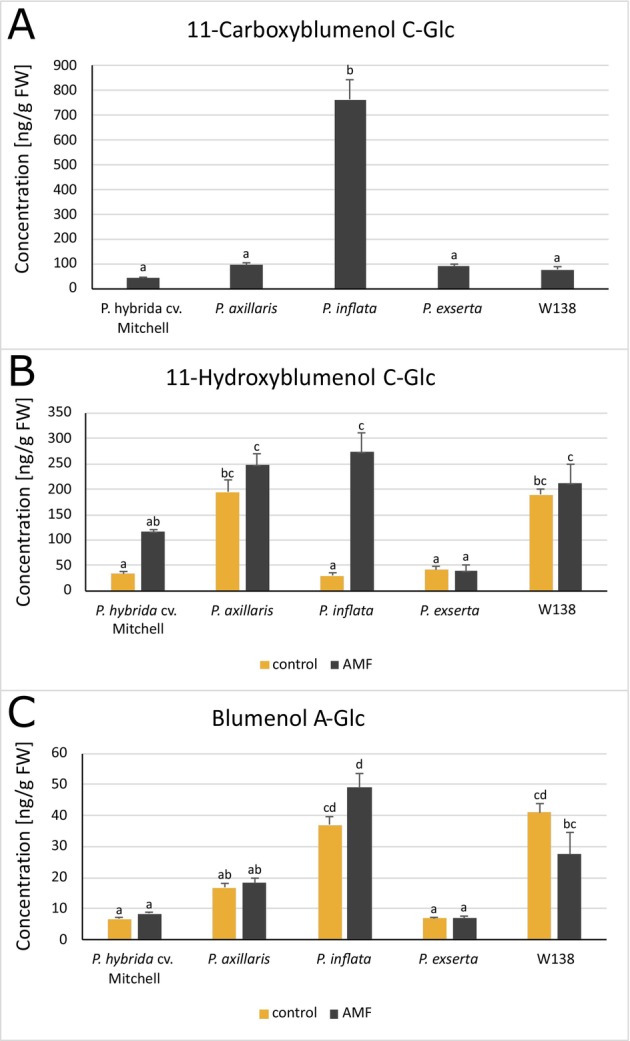
Concentration of the blumenol derivatives 11‐Carboxyblumenol C‐Glc (A), 11‐Hydroxyblumenol C‐Glc (B), and Blumenol A‐Glc (C) in leaves of five *Petunia* genotypes inoculated with *R*. *irregulare* (AMF) or mock (control). Different letters indicate significant differences (*p* ≤ 0.05) between the genotypes according to Dunnett's test; error bars indicate the SEM, n = 5.

Three different blumenols, namely 11‐carboxyblumenol C‐Glc, 11‐hydroxyblumenol C‐Glc, and blumenol A‐Glc, were analysed in leaves of *P. hybrida*, *P. axillaris*, *P. exserta*, *P. inflata*, and the high‐copy number *dTPH1* transposon insertion line W138 (Fig. 7). Solely 11‐carboxyblumenol C‐Glc was expressed after inoculation with AMF. Its concentration was highest in *P. inflata* (>700 ng g^−1^ FW) (Fig. 7A). 11‐hydroxyblumenol C‐Glc was detected in control and AMF samples of all five genotypes (Fig. 7B). Its concentration increased after inoculation with AMF in all genotypes, except in *P. exserta*. *P. inflata* showed the largest induction, whereas baseline levels were highest in *P. axillaris* and W138. Blumenol A‐Glc was present in all samples, with significant increases only in *P. inflata* and decreases in W138 (Fig. 7C).

### Phosphorus content and phosphate transporter gene expression

Phosphorus (Pi) content differed among species and treatments (Fig. S3). Control plants of *P. hybrida*, *P. axillaris*, and *P. exserta* showed similar Pi levels, but AMF inoculation increased Pi only in *P. axillaris*. For *P. inflata* and W138, analysis was not possible due to limited biomass.

Expression of the phosphate transporter genes *PhPT4* (mycorrhiza‐specific) and *PhPT5* (mycorrhiza‐enhanced) was measured in roots (Fig. S4). *PhPT4* was undetectable in controls and strongly induced by AMF, whereas *PhPT5* showed weak constitutive expression. Differences among species were minor, and both genes showed significant mycorrhiza‐dependent induction, consistent with previous findings (Wegmüller *et al*. 2008; Tan *et al*. 2012).

## DISCUSSION

Our results demonstrate that *Petunia*'s AM‐responsiveness to *Rhizoglomus irregulare* colonization is highly variable and influenced by both genetic and environmental factors. Among the four *Petunia* species, *P. axillaris* showed a strong positive response in leaf biomass upon mycorrhizal inoculation, whereas *P. inflata* and *P. exserta* showed reduced leaf growth under the same conditions. *P. hybrida* exhibited a mild growth promotion in leaves. These contrasting outcomes highlight the mutualism–parasitism continuum often seen in plant–AMF interactions (Smith *et al*. 2010). The transposon insertion line W138 showed no significant growth differences between inoculated and control plants, except reduced flower and capsule formation in mycorrhized plants (Suppl. Table 1). Its low vesicle formation (Fig. 2A) suggests a physiological limitation of AMF development, possibly linked to its high‐copy *dTPH1* background, which has yielded mutants impaired in mycorrhizal symbiosis (Reddy *et al*. 2007).

### Interspecific variation and physiological interpretation

Contrary to earlier studies reporting positive growth responses in *P. hybrida* under phosphate limitation (Hayek *et al*. 2012; Nouri *et al*. 2014), our plants showed mostly neutral or negative responses (Figs. 1A–E and 2). Although *P. hybrida* originates from *P. axillaris × P. inflata*, its response pattern did not resemble *P. axillaris* more closely, suggesting dominance of *P. inflata*‐like traits.

*P. exserta* showed accelerated flowering (Fig. 1D) and unchanged phosphorus uptake (Fig. S3), suggesting a carbon drain effect promoting early reproduction. In contrast, *P. hybrida* delayed flowering under mycorrhization, consistent with AMF‐induced reallocation of resources toward vegetative growth (Qu *et al*. 2021). Such AMF‐mediated phenological shifts could be exploited in ornamental breeding for early flowering.

Mycorrhization increased leaf:stem ratios in *P. axillaris* and *P. inflata* but not in *P. exserta* (Fig. 1E). Similar effects were observed for plants colonized by endophytic *Serendipita* species, enhancing photosynthetic capacity through altered biomass allocation (de Rocchis *et al*. 2022). Interestingly, AMF reduced root weight but not length (Fig. S1), indicating altered root architecture rather than growth inhibition. Comparable mycorrhizal‐induced shifts from coarse to fine roots have been reported in trifoliate orange (Yao *et al*. 2009). Enhanced water retention by AMF (Pauwels *et al*. 2023) might also explain reduced root biomass, as plants require fewer roots to access water.

### Colonization, phosphorus uptake, and transporter expression

Colonization parameters revealed full root colonization and high arbuscule abundance in *P. axillaris* but reduced colonization in *P. exserta* (Fig. S2). Despite arbuscule formation and symbiotic gene induction (Fig. S4), *P. exserta* showed no phosphorus uptake increase, whereas *P. axillaris* displayed strong Pi accumulation (Fig. S3). Phosphorus levels correlated with AMF colonization parameters (Table S2), indicating efficient mycorrhizal Pi transfer in *P. axillaris* (Bucher 2007; Bucher *et al*. 2014).

Roots of the transposon insertion line ‘W138’ had slightly lower colonization and negligible vesicle abundance, suggesting impaired fungal development. Nonetheless, both *PhPT4* and *PhPT5* were upregulated by AMF (Fig. S4), confirming functional signalling. *PhPT4* was strictly AMF‐induced, while *PhPT5* showed weak basal expression, consistent with previous results (Wegmüller *et al*. 2008; Tan *et al*. 2012). All genotypes thus activated the mycorrhizal Pi‐uptake pathway, but in *P. exserta*, colonization efficiency likely limited nutrient benefit. Together, these patterns indicate that *P. axillaris* effectively translates colonization into growth, whereas *P. exserta* is largely AMF‐unresponsive, which highlights contrasting AMF symbiotic strategies. This contrast in responsiveness forms the basis for selecting *P. axillaris* and *P. exserta* as parental lines in a RIL population to uncover the genetic determinants of AM‐responsiveness.

### Quantitative nature and genetic diversity of AM‐responsiveness

Trial 2 confirmed that AM‐responsiveness is a quantitative trait. The 19 RILs displayed transgressive segregation, ranging from negative to strongly positive shoot responses (Fig. 1F). Some RILs (e.g. 147, 145, 155) outperformed *P. axillaris*, while others resembled *P. exserta*. ANOVA confirmed significant genotypic differences (Fig. 1F), indicating heritable variation suitable for QTL mapping. The observed range of responsiveness is consistent with other species (Stahlhut *et al*. 2023). The RILs showed no segregation distortion toward either parental allele, unlike findings by Guo *et al*. (2017). Transgressive RILs (e.g. RIL147) likely carry novel allele combinations conferring superior symbiotic performance.

Although root and shoot biomass were strongly correlated (Fig. S5), colonization intensity did not correlate with biomass or benefit, confirming that colonization alone is not predictive of AM effect (Sawers *et al*. 2017; Watts‐Williams *et al*. 2019; Servanté *et al*. 2024).

Expanding phenotyping to all 173 RILs (Warner & Walworth 2010; Guo *et al*. 2017) would allow QTL mapping for biomass traits data to identify candidate genes. Integration with transcriptomic data could further identify differentially expressed genes following AMF inoculation, especially when sampled at vegetative and generative stages. Such expression differences might explain the varied outcomes observed between the first and the second trial due to differences in harvest timing.

Subsequent validation via targeted mutagenesis (Chopy *et al*. 2020), or GWAS across broader *Petunia* germplasm (Strazzer *et al*. 2023) could identify markers for breeding AM‐responsive cultivars. Alternatively, insertion mutagenesis using the *dTPH1* transposon in the W138 background is feasible (Gerats & Vandenbussche 2005).

### Environmental dependency and G × E interactions

Trials 1 and 2 revealed inconsistent responses, particularly in *P. exserta*, where AMF effects varied strongly (Fig. 1A,F). *P. axillaris* showed consistent AMF/control ratios across trials, indicating stable AM‐responsiveness and limited genotype × environment interaction, whereas *P. exserta's* strong shift in AM‐responsiveness suggests greater environmental sensitivity. Since the inoculum source was constant (same production batch, C. Schneider, INOQ GmbH, pers. comm.), these differences must reflect environmental factors: Trial 2 had four extra weeks of cultivation, 1.7°C lower average temperature, 2.3× higher PAR and 19.4% higher relative humidity. These variables appear to overshadow genetic effects in some cases, particularly for *P. exserta*.

The three‐environment experiment (Trial 3) confirmed strong G × E interactions (Fig. 4). Some traits (e.g. leaf and stem weights) lacked main genotype effects, indicating plasticity. Others, like shoot length and total biomass, had strong genotype and G × E effects, implying more stable genetic control. In Experiment 2 (high light, long photoperiod), nearly all RILs exhibited positive AMF effects (>100%), whereas in Experiments 1 and 3 (lower light/shorter photoperiod), AMF suppressed growth (Fig. 3). Thus, the AMF mutualism shifts toward parasitism under carbon‐limiting conditions (Smith *et al*. 2010). Genotype‐specific responses varied with environment—RILs genetically closer to *P. axillaris* performed worse in Exp. 1 but better in Exp. 2 (Fig. 5). Significant G × E interactions across most traits confirm that AM‐responsiveness depends on both genetic and environmental factors.

Colonization traits were also affected by G × E: F%, M%, A%, and V% differed strongly across experiments (Fig. 4B). Genotype effects were strongest for M% and A%, consistent with host control of arbuscule development via the Common Symbiosis Signalling Pathway (Carbonnel & Gutjahr 2014). Reduced colonization in Exp. 3 corresponded to shorter photoperiod and lower photosynthate availability. PCA confirmed clear environmental separation of colonization data but overlapping harvest data (Fig. 6), indicating that colonization is more genotype‐dependent, while biomass traits are more environment‐driven.

### Blumenols as metabolic markers

Three blumenols were analysed as shoot biomarkers of AMF colonization. 11‐carboxyblumenol C‐Glc occurred exclusively in AMF‐inoculated plants, confirming its reliability as a presence marker (Mindt *et al*. 2019). However, its concentration did not correlate with colonization intensity (Table S2), suggesting limited quantitative value, unlike earlier reports (Wang *et al*. 2018). 11‐hydroxyblumenol C‐Glc and blumenol A‐Glc were detected in both control and inoculated samples but increased with AMF in most genotypes, except *P. exserta*. *P. inflata* showed the highest blumenol levels, possibly reflecting strong carbon allocation to symbiosis despite growth suppression. In contrast, *P. exserta* displayed no increase, consistent with weak colonization. Detectable blumenols in AMF‐free controls (notably *P. axillaris* and W138) may suggest AM‐independent production, thus precluding their use as metabolic AMF colonization markers. The positive correlation between blumenols and phosphorus levels may result from shared apocarotenoid precursors with strigolactones (Fiorilli *et al*. 2019). Thus, 11‐carboxyblumenol C‐Glc serves as a robust colonization indicator, while 11‐hydroxyblumenol C‐Glc reflects nutrient‐related metabolic activity.

### Implications for breeding and environmental optimization

Our results highlight that AMF effects on plant performance depend on complex genotype × environment interactions. Breeding for AM‐responsiveness must therefore validate QTL across multiple environmental contexts. Environment‐specific QTL analysis, followed by overlap filtering, may identify stable loci (Galván *et al*. 2011).

Alternatively, optimizing environmental factors can enhance AMF benefits independently of genotype. This is especially relevant for greenhouse ornamentals, where light and photoperiod can be controlled. As shown in Exp. 2, favourable light regimes can induce a genotype‐independent positive response (Fig. 3). Systematic testing of environmental conditions could identify optimal settings for maximizing AMF‐derived growth benefits.

In conclusion, *Petunia* is an effective model for analysing the genetic and environmental regulation of AM symbiosis. Our data demonstrate that colonization per se does not ensure growth promotion—benefits depend on arbuscule function, nutrient exchange and light availability. Clear genetic variation exists for colonization intensity and AM‐responsiveness, indicating breeding potential. Metabolic (blumenols) and molecular (phosphate transporter) markers proved valuable for confirming symbiosis activity, even when phenotypic effects were subtle. These tools will facilitate future studies on AMF interactions and enable breeding or environmental optimization for improved symbiotic performance in *Petunia* and related species.

## AUTHOR CONTRIBUTIONS

JB and PF designed the research, interpreted the data, and wrote the manuscript. JB carried out Trial 1 and Trial 3, and KF carried out Trial 2. RH analysed the content of blumenol derivatives. JB conducted all statistical analyses. All authors contributed to the article and approved the submitted version.

## FUNDING INFORMATION

All the work was funded by the Thuringian Ministry of Economics, Science and Digital Society except the implementation of the second experiment, which was funded by the Ministry of Consumer Protection, Food and Agriculture of the Federal Republic of Germany and the Ministry for Science, Research and Culture of the State of Brandenburg.

## CONFLICT OF INTEREST STATEMENT

The authors declare that the research was conducted in the absence of any commercial or financial relationships that could be construed as a potential conflict of interest.

## Supporting information


**Data S1.** Supporting Information.

## Data Availability

All data generated or analysed during this study are provided in this manuscript and its Supporting Information S1 files or it will be provided upon a reasonable request.
